# Scale-Up Preparation of Crocins I and II from *Gardenia* *jasminoides* by a Two-Step Chromatographic Approach and Their Inhibitory Activity Against ATP Citrate Lyase

**DOI:** 10.3390/molecules26113137

**Published:** 2021-05-24

**Authors:** Shuguang Guan, Qiaoli Pu, Yinan Liu, Honghong Wu, Wenbo Yu, Zifeng Pi, Shu Liu, Fengrui Song, Jingya Li, De-An Guo

**Affiliations:** 1 College of Pharmacy, Changchun University of Chinese Medicine, Changchun 130117, China; gsgwww@126.com (S.G.); Puqiaolily@163.com (Q.P.); a511cs@126.com (W.Y.); 2 Changchun Institute of Applied Chemistry, Chinese Academy of Sciences, Changchun 130022, China; mslab20@ciac.ac.cn (S.L.); songfr@ciac.ac.cn (F.S.); 3 Shanghai Institute of Materia Medica, Chinese Academy of Sciences, Shanghai 201203, China; ynliu@simm.ac.cn (Y.L.); jyli@simm.ac.cn (J.L.); 4 University of Chinese Academy of Sciences, Beijing 100049, China; s18-wuhonghong@simm.ac.cn; 5 Changchun Sunnytech Co., Ltd., Changchun 130061, China

**Keywords:** crocins, preparation method, macroporous resin column chromatography, high-speed counter-current chromatography, inhibitory activity on ATP citrate lyase

## Abstract

Crocins are highly valuable natural compounds for treating human disorders, and they are also high-end spices and colorants in the food industry. Due to the limitation of obtaining this type of highly polar compound, the commercial prices of crocins I and II are expensive. In this study, macroporous resin column chromatography combined with high-speed counter-current chromatography (HSCCC) was used to purify crocins I and II from natural sources. With only two chromatographic steps, both compounds were simultaneously isolated from the dry fruit of *Gardenia jasminoides*, which is a cheap herbal medicine distributed in a number of countries. In an effort to shorten the isolation time and reduce solvent usage, forward and reverse rotations were successively utilized in the HSCCC isolation procedure. Crocins I and II were simultaneously obtained from a herbal resource with high recoveries of 0.5% and 0.1%, respectively, and high purities of 98.7% and 99.1%, respectively, by HPLC analysis. The optimized preparation method was proven to be highly efficient, convenient, and cost-effective. Crocins I and II exhibited inhibitory activity against ATP citrate lyase, and their IC_50_ values were determined to be 36.3 ± 6.24 and 29.7 ± 7.41 μM, respectively.

## 1. Introduction

Crocins are highly valuable natural products that have been reported to treat human disorders, such as multiple sclerosis [1], depressive disorder [2,3], inflammatory diseases [4], ophthalmic disease [5], and cancer [6]. It has also been reported that crocins show biological functions, such as neuroprotective effects [7], anti-inflammatory activities [8], neurodegenerative diseases [9], allergic airway asthma [10], cardioprotective effects [11], counteracting performance deficits [12], potential therapy for Alzheimer’s disease [13], retinal ganglion cell protection [14], platelet apoptosis protection and platelet aggregation inhibition [15], and demyelination and neurodegeneration prevention [16], in animal models. In addition, as kinds of colored apocarotenoids, crocins are often used as valuable spices and colorants in the food industry [17,18]. They originate from the stigmas of *Crocus sativus* (saffron), which has been reported to be the most expensive spice and colorant in the world. Due to the limitation of obtaining this type of compound, the commercial prices of the two major crocins—crocins I and II—are extremely expensive.

Though synthetic biology methods [17,19] have been attempted to be used to produce crocins I and II, isolation from plant resources is still the only method for their large-scale production. However, except for saffron, only a few distantly related plants, such as *Gardenia jasminoides* (which grows in many countries) [17], produce crocins. Hence, taking full advantage of the cheap fruits of *G. jasminoides* to obtain crocins I and II remains high in demand.

The successful isolation of compounds from *G. jasminoides* using HSCCC has been previously reported [20,21,22,23]. The preparation of geniposide [20,21] and the diversity-oriented isolation of *G. jasminoides* [22,23] have been claimed. Considering isolation time, solvent consumption, extract efficiency, etc., the isolation procedures recorded in the literature have not been economical and efficient enough for the large-scale preparation of crocins I and II.

Abundant research concerning the biological and pharmacological properties of crocin has been conducted. It has been shown that crocin exhibits beneficial effects on lipid metabolism. However, the mechanism of how crocin improves lipid metabolism is elusive [24,25,26]. ATP citrate lyase (ACLY) is a cytosolic enzyme that catalyzes the cleavage of citrate into acetyl-CoA and oxaloacetate. It is abundantly expressed in lipogenic tissues in nonruminating mammals, so it plays a pivotal role in supplying acetyl-CoA for both cholesterogenesis and lipogenesis [27]. It is also important in females for the systemic handling of dietary carbohydrates and the preservation of metabolic homeostasis [28]. ACLY has been validated as a drug target for the treatment of hypercholesterolemia, and it is considered to be a potential target in the prevention of fatty liver and diabetes diseases.

In the present work, we successfully established a practical method for the simultaneous preparation of crocins I and II at a large scale and with high purity, high recovery, high efficiency, and low cost. Moreover, crocins I and II were confirmed to inhibit ACLY.

## 2. Results and Discussion

### 2.1. The Identification of the Two Target Compounds

Compounds **1** and **2** purified from HSCCC were identified as crocins I and II, respectively, on the basis of their MS, 1H-NMR, and 13C-NMR spectra (Please see Supplementary Materials); all data were in accordance with those reported in literature [23].

### 2.2. The Key Steps in the Selection of Fractionation Approach

Accounting for the high polarity characteristic of crocins, the classical silica gel column chromatography and liquid–liquid solvent extraction methods were not considered in the isolation steps in order to avoid yield loss. Instead, macroporous resin column chromatography was selected for the first fractionation of the total fruit extract of *G. jasminoides*.

The concentration and total amount of the loaded sample were screened based on the reported methods [20,23,29]. In this case, however, macroporous resin column plugging was observed if the concentration of the loaded sample was above 1 g/50 mL. Thus, several concentrations of the loaded sample were investigated (Table 1). The ratio of the weight of crude extracts and the volume of water was finally selected as 1:60 (g/mL, *w*/*v*).

### 2.3. The TLC Results of the Eluents from the Macroporous Resin Column Chromatography

**Figure 1:**
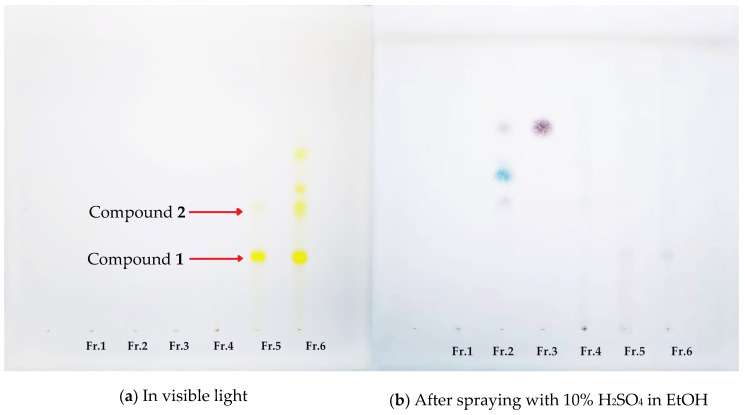
The TLC chromatograms of Fr.1–Fr.6 eluted from macroporous resin column chromatography (Developing solvents: CHCl_3_-MeOH-HCOOH-H_2_O/5:2:0.5:0.5; sample volume: 10 μL; sample concentration: 2 mg/mL).

**Figure 2:**
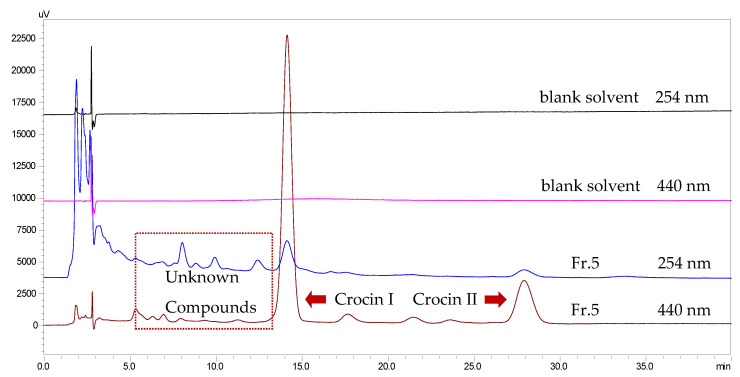
The HPLC chromatograms of Fr. 5.

The eluents obtained from the macroporous resin column chromatography were collected every 600 mL. TLC was used to detect the main components in each elution. The last eight 600 mL elutions (4800 mL of Fr. 5) eluted by 45% EtOH were combined because of two main spots (crocins I and II) being observed (both in visible light and after spraying with 10% H_2_SO_4_ in EtOH while being heated at 105 °C) on the TLC plate (Figure 1). This is also a key necessary skills for getting high purities of crocins I and II in only two isolation steps. The HPLC chromatograms of Fr. 5 are shown in Figure 2. 

### 2.4. Selection of the Two-Phase Solvents by Settling Time and Separation Factors

Five kinds of two-phase solvents were screened according to the settling times of the two-phase solvents and the preparation factors (Table 2). As a result, the No.2 solvent system was selected because it showed an acceptable settling time (17 sec) for the two-phase solvents and a good separation factor (**α**). Even though the *K_D_* value (0.11) of crocin I was too low compared the expected *K_D_* value (around 1), the separation efficiency was not affected in this case because the eluents from macroporous resin column chromatography were carefully collected through the TLC-presented spots.

### 2.5. Forward and Reverse Rotations Successively Applied in the HSCCC Isolation Procedure

**Figure 3:**
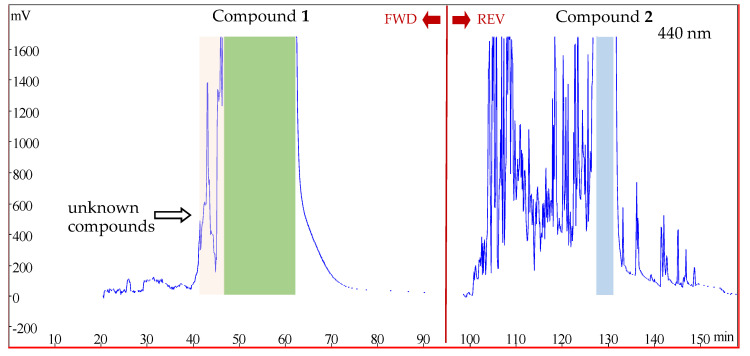
HSCCC purification of crocins I and II from the crocin-containing fraction by forward and reverse rotation.

Crocins I and II were purified from HSCCC by successively using forward and reverse rotations in a single run (Figure 3). After a forward HSCCC rotation for about 70 min, target compound **1** was completely eluted out of the column. In order to save time and solvents, a reverse rotation was applied at about 95 min, and target compound **2** was totally eluted out within 40 min.

The measuring wavelength selected at 440 nm for HSCCC isolation was mainly because of the following considerations. There was less crocin II than crocin I, so crocin II was considered more in the separation process. The impurities between crocins I and II in both HPLC and HSCCC chromatograms could only be observed at 440 nm, and the purity of crocin I, which was also separated at 440 nm, could be guaranteed by collecting the highest peak due to its relatively high amount.

For the follow-up preparation, the reverse rotation of HSCCC could be operated earlier at around 80 min, and then another 15 min could be saved

### 2.6. The Recoveries and the Purities of the Two Target Compounds

From 15 mL of crocin-containing sample solution (300 mg of Fr. 5), 56.5 mg of crocin I and 11.1 mg of crocin II were purified by HSCCC with recoveries of 18.8% and 3.7%, respectively.

From 40 g of the total fruit extract of *G. jasminoides,* 3.4 g of Fr. 5 were obtained through macroporous resin column chromatography with a recovery of 8.5%.

From 500 g of the dry fruit powder of *G. jasminoides*, the 147.9 g of the total fruit extract of *G. jasminoides* was obtained with a recovery of 29.6%.

**Figure 4:**
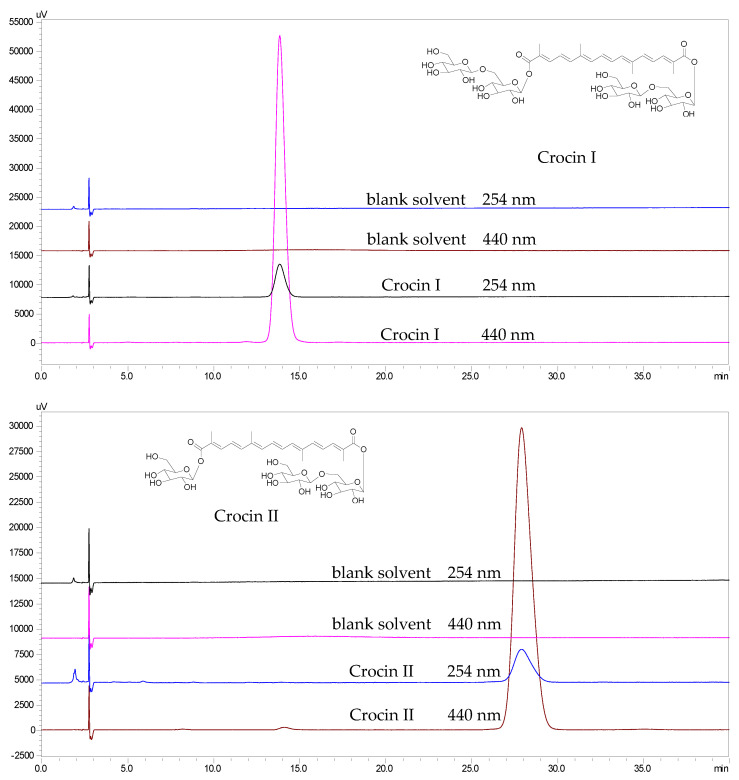
The HPLC chromatograms of crocins I and II after purification by HSCCC.

From the above-mentioned recoveries, crocins I and II were yielded at high purities of 98.7% and 99.1% (Figure 4), respectively, from the dry fruit powder of *G. jasminoides* with high recoveries of 0.5% and 0.1%, respectively, by just two-step chromatography.

### 2.7. Inhibitory Activity of Compounds on ACLY

**Figure 5:**
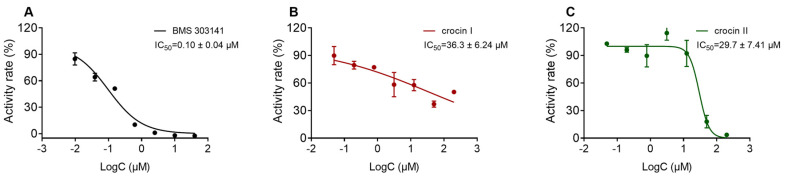
Inhibitory curves of crocins I and II on ACLY. (**A**) Effect of positive control BMS 303141. (**B**,**C**) Effects of compounds **1** (crocin I) and **2** (crocin II), respectively, on the catalytic activity of ACLY with multiple concentrations.

The primary screen at a single dose of 2 mM indicated that crocins I and II had >90% inhibition. Crocins I and II were then diluted to a 1:4 ratio to generate eight concentrations ranging from 200 to 0.048 µM to determine their IC_50_ values. Thus, the two compounds were confirmed to inhibit ACLY, and the known ACLY inhibitor BMS 303141 was a positive control. The IC_50_ values of crocin I (compound **1**) and crocin II (compound **2**) were determined to be 36.3 ± 6.24 and 29.7 ± 7.41 μM, respectively (Figure 5).

## 3. Materials and Methods

### 3.1. Reagents

For isolation, ethanol (EtOH), n-butanol (n-BuOH), ethyl acetate (EtOAc), sulphuric acid (H_2_SO_4_) and other used organic solvents were all of analytical grade and purchased from Beijing Chemical Works (Beijing, China). For HPLC analysis, methanol (MeOH) was chromatographic grade and obtained from Thermo Fisher Scientific Inc. (Waltham, MA, USA), and purified water was purchased from Hangzhou Wahaha Group Co., Ltd. (Hangzhou, China). For the activity assay, an ADP-Glo™ Kinase Assay kit was obtained from Promega Corporation (Madison, WI, USA), BMS 303141 was obtained from MedChemExpress (Monmouth Junction, NJ, USA), ACLY (His) was from Sino Biological Inc. (Beijing, China), and DMSO/D8418 was purchased from Sigma-Aldrich (St. Louis, MO, USA).

### 3.2. Materials

The dry fruits of *G. jasminoides* were purchased from Hongjian medicinal materials company of Jilin province (China) and were identified by Professor Jinglei Xiao (Changchun University of Chinese Medicine). The voucher sample was stored in the natural product laboratory of Changchun University of Chinese Medicine.

D101 macroporous resins were obtained from Chemical Plant, Nankai University, Tianjin (China).

Type G silica gel plates were from Qingdao Ocean Chemical Plant Branch, Qingdao (China).

### 3.3. Apparatus

The HSCCC purification was performed on a TBE-300C instrument (Tauto Biotechnique Company, Shanghai, China) consisting of a 300 mL PTFE multilayer coil (the size of the PTFE tubing: 1.9 mm id and 2.9 mm od), equipped with a TBP-5002 constant-temperature circulating implement, an HW-2000 chromatographic station, and a TBE-2000 UV detector. Additionally, a PTFE 20 mL sample loop was used. The HPLC analysis was accomplished with a Prominence-LC-20AT Series high-performance liquid chromatograph equipped with a Prominence Diode Array Detector SPD-M20A (Shimadzu Corporation, Kyoto, Japan). The Zorbax SB C18 reversed-phase column (250 × 4.6 mm id, 5 μm, Agilent Technologies) was utilized to analyze the purities of crocins I and II. MS analyses were performed on an Agilent 6545 Accurate-Mass Q-TOF LC/MS system (Agilent Technologies, Santa Clara, CA, USA). The NMR spectra were obtained from a Bruker Avance II 400 MHz NMR spectrometer (Bruker Corporation, Billerica, MA, USA). The reaction plates were read on a PerkinElmer EnVision plate reader (PerkinElmer, MA, USA).

### 3.4. Selection of the Concentration and the Amount of Loaded Sample on the Macroporous Resin Column Chromatography

In a bid to obtain a better isolation efficiency through macroporous resin column chromatography, the best concentration and total amount of the loaded sample were investigated based on methods reported in the literature [20,23,29]. The concentration and amount of the loaded sample were modified by the experiments. First, 1.02 g of crude extracts was added to 5 mL of water and completely stirred. Insoluble substances were observed in the mixed solution. Then, another 5 mL of water were added to the mixed solution and successively stirred completely to make the volumes of the mixed solution 10, 15, 20, 25, and 30 mL in turn until the insoluble substances were not observed. The maximum concentration of the sample was determined to be about 1 g/30 mL. Then, 100 g of the D101 macroporous resin in water were loaded into a glass column (diameter of 3 cm and bed height of 20 cm). The moderate amount of crude extracts was dissolved in different volumes of water (the maximum concentration was 1 g/30 mL) to make sample solutions, and then the sample solutions were successively loaded in the D101 macroporous resin columns. The eluents were collected per 10 mL, and the overload of the macroporous resin was detected by TLC. The resin was identified to be overloaded if purple spots (after spraying by a 10% H_2_SO_4_-EtOH solution) were observed on TLC plates. The amount of loaded sample was investigated three times (twice for 100 g of macroporous resin and once for 400 g of macroporous resin) on the macroporous resin column.

As a result, 1 g of the fruit extract of *G. jasminoides* dissolved in 60 mL of water (1 g/60 mL) was proven to be proper for isolation, and the weight ratio of the amount of the loaded sample and the macroporous resin was selected to be 1 g/25 g after being screened.

### 3.5. Preparation of Crude Sample from the Fruits of G. jasminoides

The dry fruit powder (500 g) of *G. jasminoides* was extracted twice under reflux with 5000 mL of 40% EtOH for 2 h. After filtration, the supernatant was combined and concentrated by a rotary evaporator under reduced pressure at 50 °C. The syrup was further freeze-dried, and the extract (147.9 g) was obtained. Forty grams of the extract were chromatographed on a macroporous resin column and successively eluted with water (600 mL) and 10% EtOH (600 mL) (combined to 1200 mL; 12.3 g of Fr. 1), 25% EtOH (7800 mL, collected into two fractions—8.6 g of Fr. 2 (formerly 1800 mL) and 2.8 g of Fr. 3 (later 6000 mL)), 45% EtOH (9600 mL, collected into two fractions—0.7 g of Fr. 4 (formerly 4800 mL) and 3.4 g of Fr. 5 (later 4800 mL)), and 95% EtOH (1800 mL; 0.9 g of Fr. 6) to yield six fractions. A total of 3.4 g of Fr. 5 were obtained for HSCCC purification.

### 3.6. Selection of HSCCC Solvent System

In an attempt to achieve the best purification efficiency of the target compounds in the shortest possible time, the partition coefficient (*K_D_*) and settling times were both considered in the two-phase solvent system selection. The *K_D_* values were determined by HPLC, as previously reported [30,31]. Based on the physical characteristics (high polarity and water-solubility) of the target compounds, a solvent system composed of EtOAc, n-BuOH, and water was selected, and different volume ratios were screened. As a result, the solvent system composed of EtOAc-n-BuOH-water (1:1:2) was determined to be the appropriate one.

### 3.7. Selection of the Separation Parameter of HSCCC

In addition to the screening of the two-phase solvent system for HSCCC purification procedure, other separation parameters were selected. The effects of the rotator speeds (750, 850, and 900 rpm) were firstly screened for their impacts on the retention rates (retention volume/total volume, %) of the stationary phase. At the rotator speed of 750 rpm, the retention rate was measured as 52.3%. At the rotator speed of 900 rpm (the largest speed of the used HSCCC instrument), the HSCCC chromatogram showed an instable baseline, so the rotator speed of 850 rpm was selected because of its acceptable stationary phase retention rate (64.0 %) and stable chromatogram baseline. Then, the flow rates (2.5, 5.0, and 8.0 mL/min) were assessed at a rotator speed of 850 rpm and an injection concentration of 50 mg/15 mL (lower phase) of Fr.5. When the flow rate was 8.0 mL/min, a loss of the stationary phase was observed. At the flow rates of 2.5 and 5.0 mL/min, the retention rates were good and a loss of the stationary phase was not observed. After considering the preparation speed of the target compounds, the flow rate of 5.0 mL/min was finally selected. The effects of the amount of loaded sample (100, 200, 300, and 400 mg) on the degrees of separation and the retention rates were all investigated through single-factor experiments. It was found that the large amount loaded sample (400 mg) had a poor solubility in 15 mL of the lower phase, so a 300 mg sample dissolved in 15 mL of the mobile phase was selected for one run of HSCCC separation.

### 3.8. Preparation of Two-Phase Solvent System and Sample Solution

The two-phase solvent system, as described, above was prepared by adding the solvents to a separatory funnel while complying with the volume ratios. The solvent system was thoroughly shaken and settled (for about 30 min) at room temperature. Then, the upper and the lower phases were easily separated. Both phases were separately degassed by ultrasonication for 20 min prior to use.

A 300 mg sample of Fr. 5 (for HSCCC purification fraction) was dissolved in 15 mL of the lower phase to obtain the sample solution.

### 3.9. The HSCCC Purification Procedure

After the upper phase was filled completely in the multilayer coiled column of HSCCC with the flow rate of 50 mL/min, the HSCCC instrument was turned on and the rotator speed was set at 850 rpm. Then, the lower phase was pumped into the column at a flow rate of 5 mL/min. When the equilibrium between the two phases was achieved, 15 mL of the sample solution (300 mg of Fr. 5) were injected into the HSCCC instrument through the sample port (with a 20 mL loop). The effluents from the outlet of the column were continuously monitored at 440 nm, and the column temperature was set at 25 °C. Target compound **1** was collected over 49–60 min, and compound **2** was collected over 126–132 min.

After a forward HSCCC rotation for about 70 min, a reverse rotation was applied for about 95 min. The detailed operation was as follows. When compound **1** was completely eluted out from HSCCC at the rotator speed of 850 rpm in the forward (FWD) model, the pump was turned off and the rotator speed was reduced to zero. The pump head of the constant flow pump was moved into the upper phase (the mobile phase was changed to the upper phase) and set to reverse (REV) rotation. The rotator speed remained at 850 rpm, and then the speed switch was turned on. When the rotator speed was stable, the pump was turned on, and the flow rate remained at 5 mL/min. Compound **2** was then eluted.

### 3.10. HPLC Analysis

Fr. 5 and target compounds **1** and **2** were dissolved in EtOH-water (1:1, *v*/*v*) and filtered with a 0.45 μm filter membrane. The concentrations of Fr. 5, compound **1,** and compound **2** were 2 mg/mL, 58.3 μg/mL, and 26.6 μg/mL, respectively. Then, the HPLC analysis was performed with a Zorbax SB C18 reversed-phase column (250 × 4.6 mm id, 5 μm). The HPLC conditions are as follows: the mobile phase of MeOH-water (45:55, *v*/*v*) and the injection volume (10 μL) followed the recomendations of Croci Stigma in the Pharmacopoeia of China (2015 edition) [32]. The flow rate of the mobile phase was 1 mL/min. The column temperature was 30 °C. The detection wavelengths were set at 254 and 440 nm for the iridoids and crocins, respectively, in the fruit of *G. jasminoides* [32]. The purities of the two target compounds purified by HSCCC were calculated through the proportion of their peak areas in HPLC [33].

### 3.11. ACLY Enzymatic Assay Based on ADP-Coupled Reaction

The activity of ACLY was determined by measuring the quantification of ADP amount generated in the enzymatic reaction. The phosphorylation reaction product ADP was detected using the ADP-Glo™ Kinase Assay kit (containing reagents 1 and 2). The assay was carried out in white 384-well plates at a final volume of 5 μL. The assay contained the following: 40 mM Tris (pH 8.0), 10 mM MgCl2, and 5 mM DTT. In this assay, a mixture containing 2 μL of ACLY, 2 μL of substrate (30 μM citrate, 5 μM ATP, and 50 μM CoA), and 1 μL of compound was incubated at 37 °C for 30 min. Compounds were prepared in DMSO and diluted in the assay to give a final concentration of DMSO not exceeding 2% (*v*/*v*). Secondly, 2.5 μL of reagent 1 was added to terminate the kinase reaction and deplete the remaining ATP. Afterwards, 5 μL of reagent 2 were added to convert ADP to ATP rapidly and to allow the newly synthesized ATP to be measured using a luciferase reaction, which was measured using a PerkinElmer EnVision reader. BMS 303141 was used as a positive control in the assay.

## 4. Conclusions

In this study, the highly polar, medicinal, and commercially-viable compounds crocins I and II were efficiently prepared at a large scale from the fruit of *G. jasminoides* by only two chromatography steps. For the first isolation step, a proper macroporous resin column chromatograph was used as a fractionation tool, and for the second isolation step, a modified HSCCC method was applied for compound purification. High efficiency, convenience, and low cost were considered in the preparation procedures. As a result, in the first step, the sample loss was minimized and a good fractionation effect was achieved. In the second step, 56.5 mg of crocin I and 11.1 mg of crocin II were simultaneously prepared in a single run from HSCCC within 135 min, consuming close to 1155 mL of solvents (495 mL of upper phase solvent and 660 mL of lower phase solvent). Crocins I and II were prepared with high recoveries (0.5% and 0.1%, respectively, calculated from the dry powder of the fruit of *G. jasminoides*) and high purities (98.7% and 99.1%, respectively). Crocins I and II were confirmed to possess ACLY inhibitory activity for the first time. Their IC_50_ values were determined to be 36.3 ± 6.24 and 29.7 ± 7.41 μM, respectively.

## Figures and Tables

**Table 1:** The screening of the best concentration and the amount of loaded sample on macroporous resin (100 g) chromatography.

	Concentration of Crude Extract (g/mL)	Loaded Volume (mL)	TLC Result
1 *	1 g/30 mL	50	No Spot
2 *	1 g/40 mL	80	No Spot
3 *	1 g/50 mL	160	No Spot
4	1 g/60 mL	280	Spot observed after the volume of loaded sample reached 250 mL
Final Choice	1 g/60 mL	240	/

* Macroporous resin column plugging was observed.

**Table 2:** Partition coefficient (*K_D_*) for target components and solvent settling time in several solvent systems.

	Solvent System (*v*/*v*)	Settling Time (sec)	*K_D_*		*α*
			Crocin I	Crocin II	
1 *	EtOAc-water (1:1)	45	/	/	/
2	EtOAc-n-BuOH-water (1:1:2)	17	0.11	1.13	10.27
3	EtOAc-n-BuOH-water (1:2:3)	11	0.15	1.67	11.13
4	EtOAc-n-BuOH-water (1:3:4)	13	0.27	1.90	7.04
5	n-BuOH-water (1:1)	19	0.38	2.07	5.45

* No samples were detected in the upper phase, so “/” means no value or zero.

## Supplementary Materials

The following are available online. Figure S1: 400 MHz ^1^H-NMR spectrum of compound **1** (in DMSO-*d*_6_); Figure S2: 100 MHz ^13^C-NMR spectrum of compound **1** (in DMSO-*d*_6_); Figure S3: 400 MHz ^1^H-NMR spectrum of compound **2** (in DMSO-*d*_6_); Figure S4: 100 MHz ^13^C-NMR spectrum of compound **2** (in DMSO-*d*_6_); Figure S5: ESI-MS spectrum of compound **1**; Figure S6: ESI-MS spectrum of compound **2**.

## Sample Availability

Not available.
