# Supplementary material for: Scale-Up Preparation of Crocins I and II from Gardenia jasminoides by a Two-Step Chromatographic Approach and Their Inhibitory Activity Against ATP Citrate Lyase
Source: Molecules. 2021 May 24;26(11):3137. doi: 10.3390/molecules26113137 (PMC8197369; doi:10.3390/molecules26113137)
Supplement: Supplementary file 1 [file molecules-26-03137-s001.zip › molecules-1227319-supplementary.pdf]

molecules

Supplementary Materials

Scale-up preparation of crocins I and II from *Gardenia Jasminoides* by a two-step chromatographic approach and their inhibitory activity on ATP citrate lyase

Shuguang Guan [1,2], Qiaoli Pu [1], Yinan Liu [3], Honghong Wu [4], Wenbo Yu [1], Zifeng Pi [2,5,*], Shu Liu [2], Fengrui Song [2], Jingya Li [3] and De-an Guo [1,3,*]

1 College of Pharmacy, Changchun University of Chinese Medicine, Changchun, 130117, China; gsgwww@126.com (S.G.); Puqiaolily@163.com (Q.P.); a511cs@126.com (W.Y.)
2 Changchun Institute of Applied Chemistry, Chinese Academy of Sciences, Changchun, 130022, China; mslab20@ciac.ac.cn (S.L.); songfr@ciac.ac.cn (F.S.)
3 Shanghai Institute of Materia Medica, Chinese Academy of Sciences, Shanghai, 201203, China; ynliu@simm.ac.cn (Y.L.); jyli@simm.ac.cn (J.L.)
4 University of Chinese Academy of Sciences, Beijing, 100049, China; s18-wuhonghong@simm.ac.cn
5 Changchun Sunnytech Co.,Ltd., Changchun, 130061, China
* Correspondence: mslab21@ciac.ac.cn (Z.P.); daguo@simm.ac.cn (D.G.); Tel.: +86-21-50271516; Fax: +86-21-50271516 (D.G.)

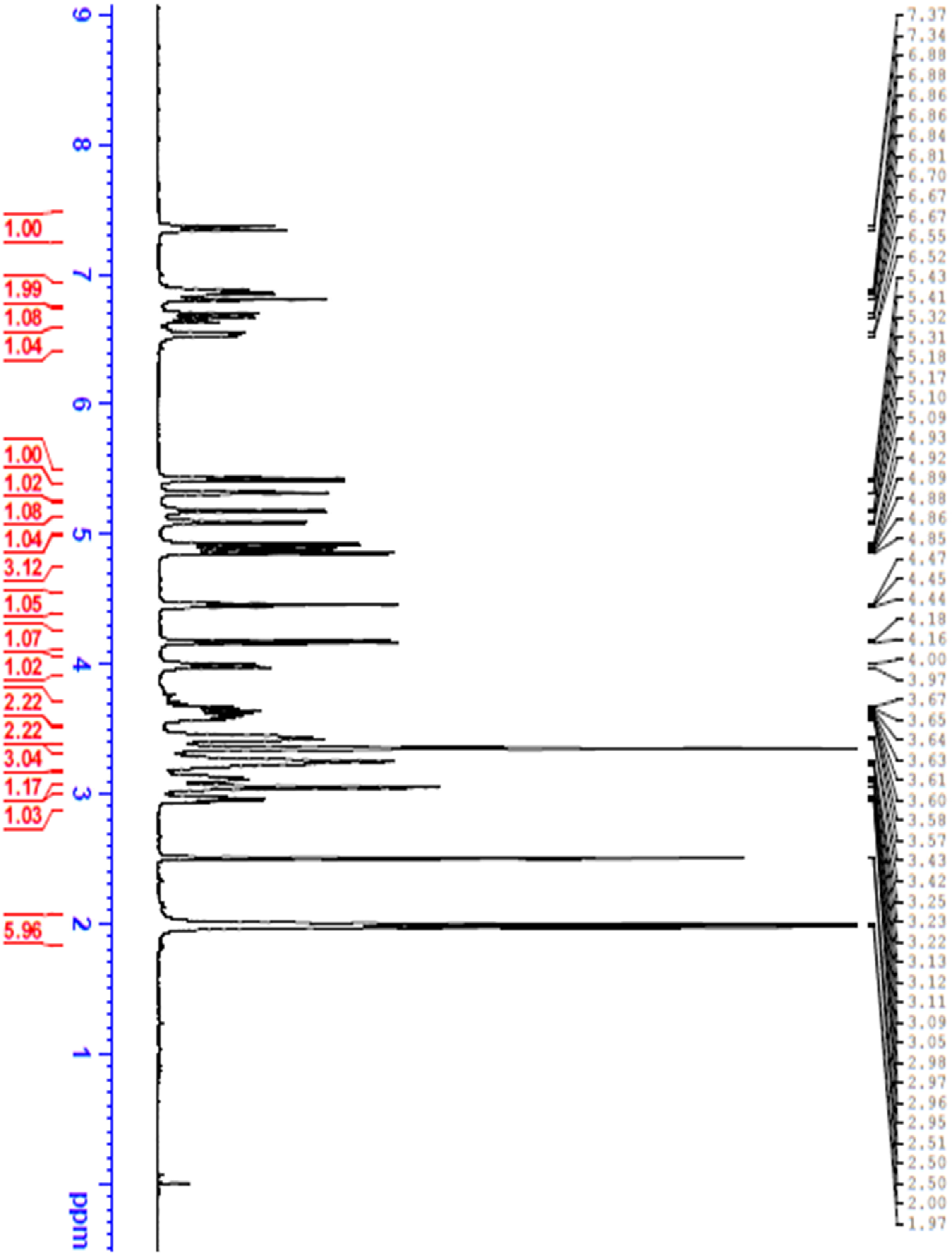

Figure S1. 400 MHz ^{1}H-NMR spectrum of compound **1**（in DMSO-d_6）.

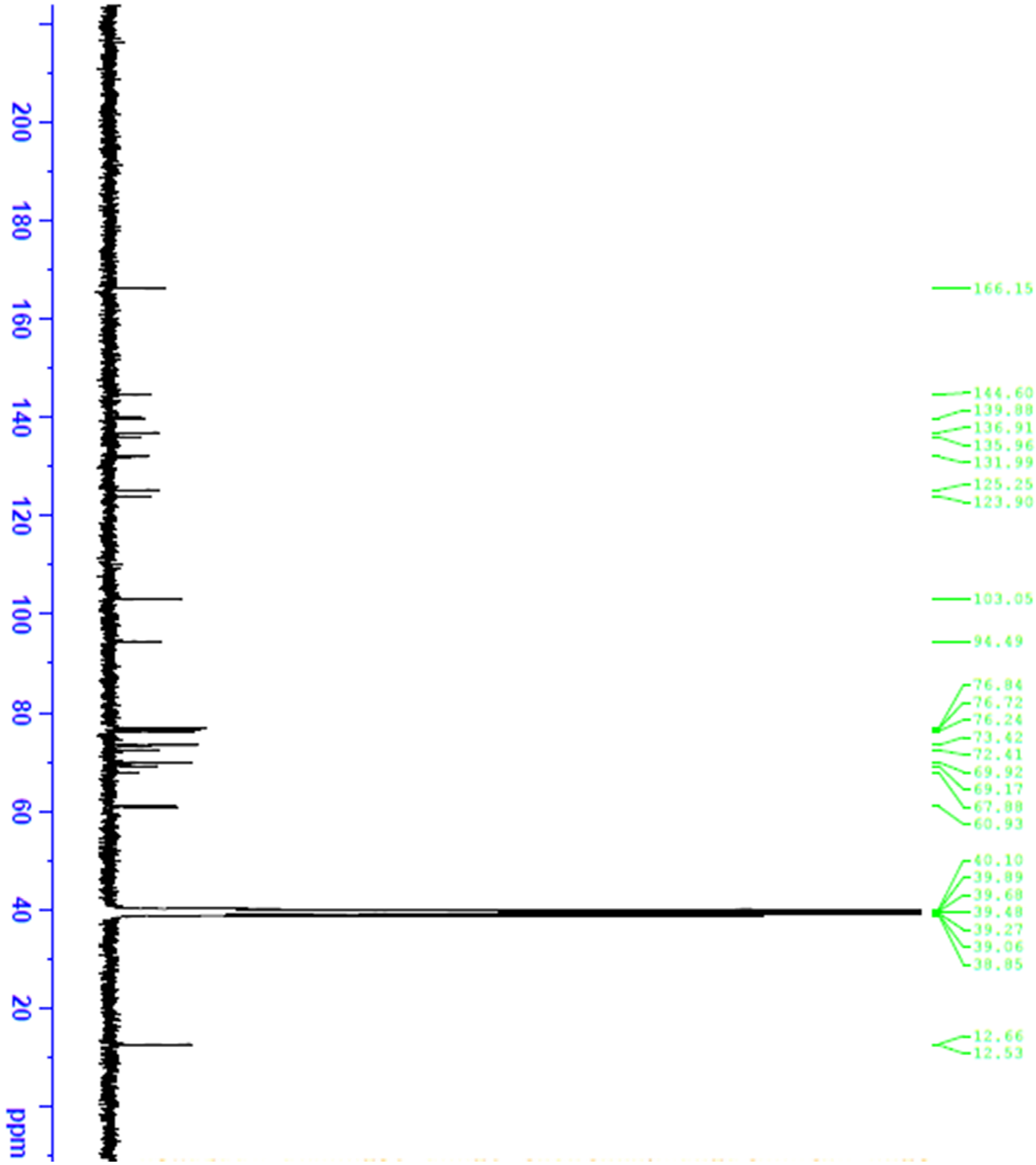

Figure S2. 100 MHz ^{13}C-NMR spectrum of compound **1 (**in DMSO-*d*$_6$)

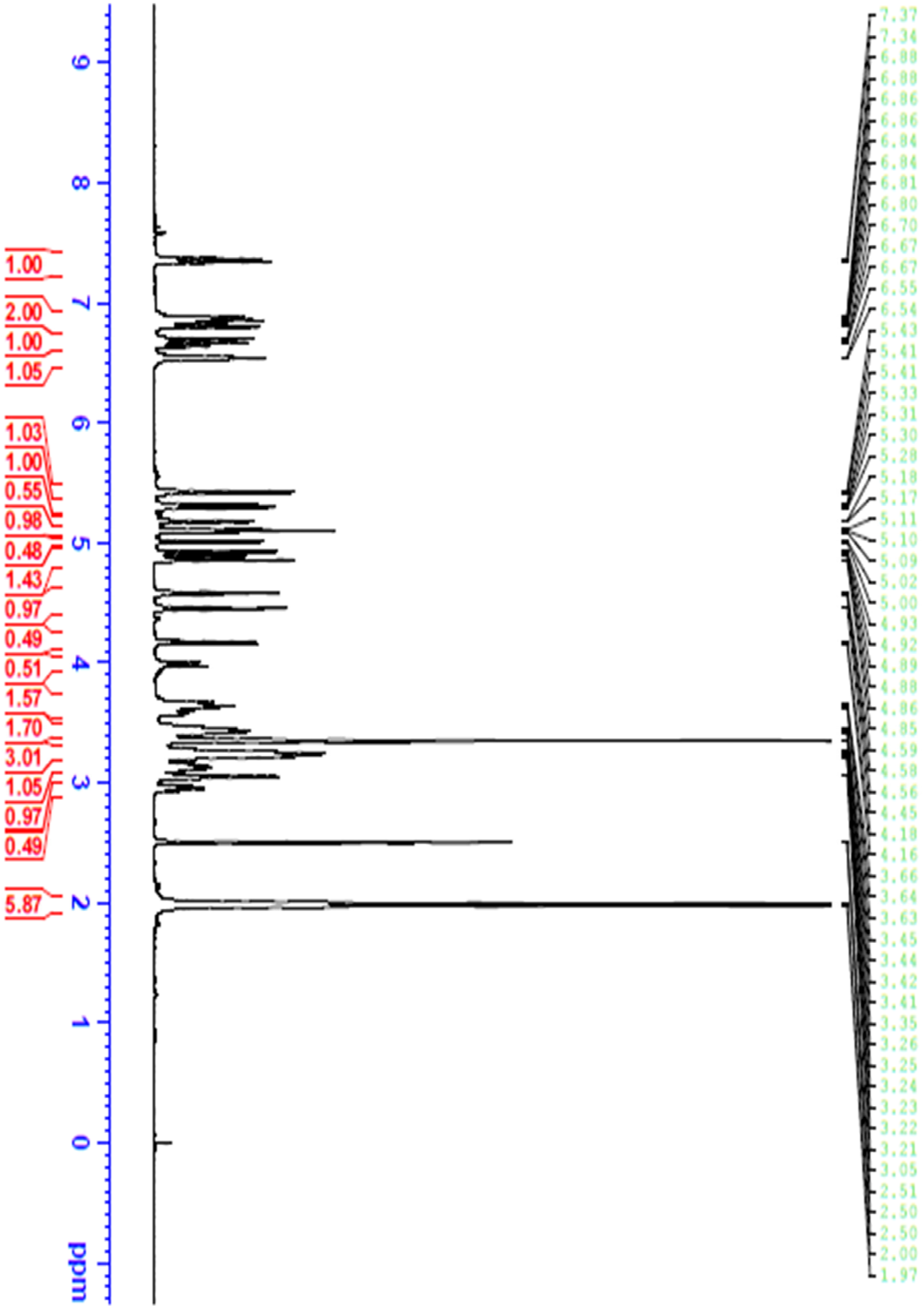

Figure S3. 400 MHz ^{1}H-NMR spectrum of compound **2** (in DMSO-d_6)

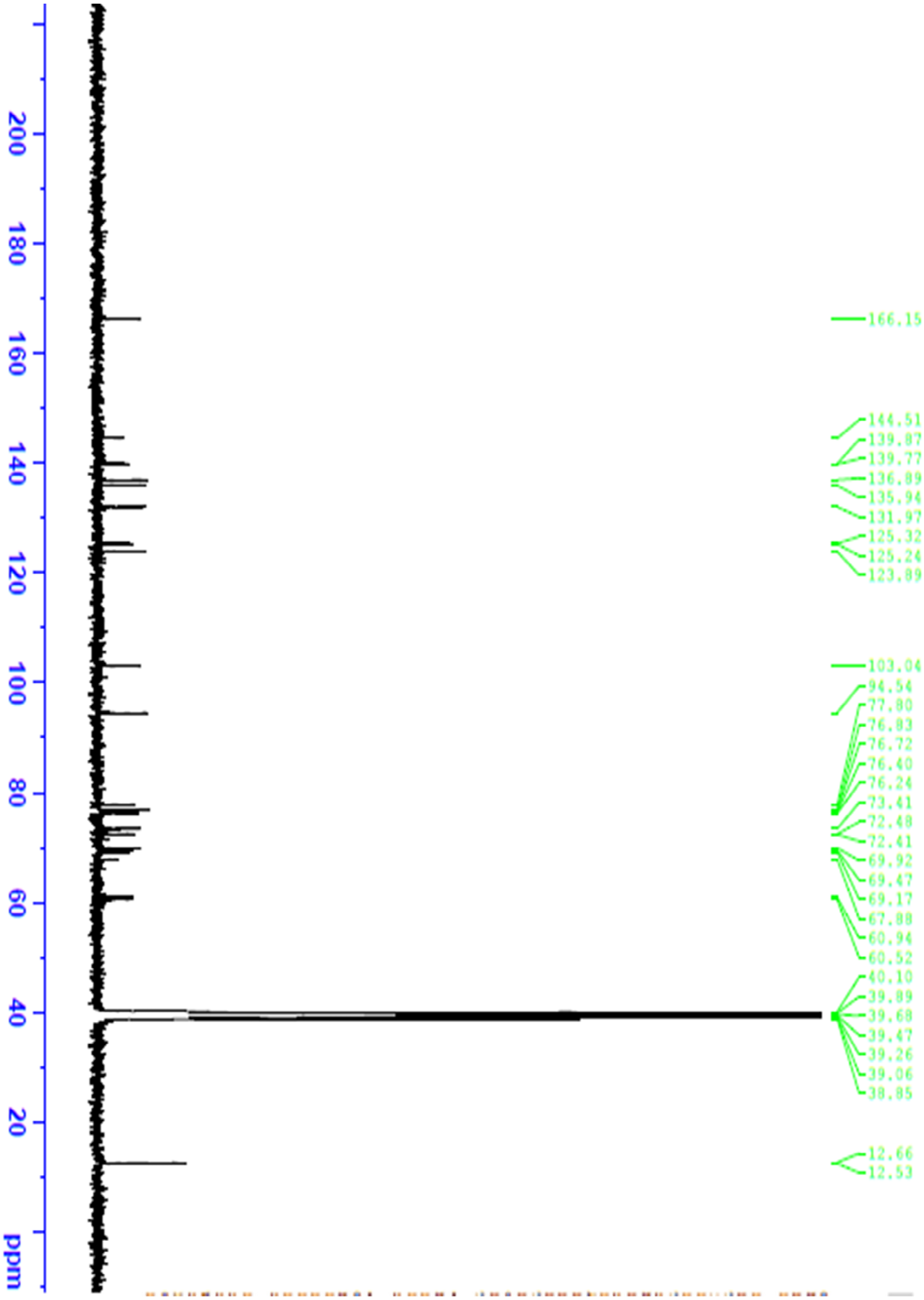

Figure S4. 100 MHz ^{13}C-NMR spectrum of compound **2** **(**in DMSO-d_6）.

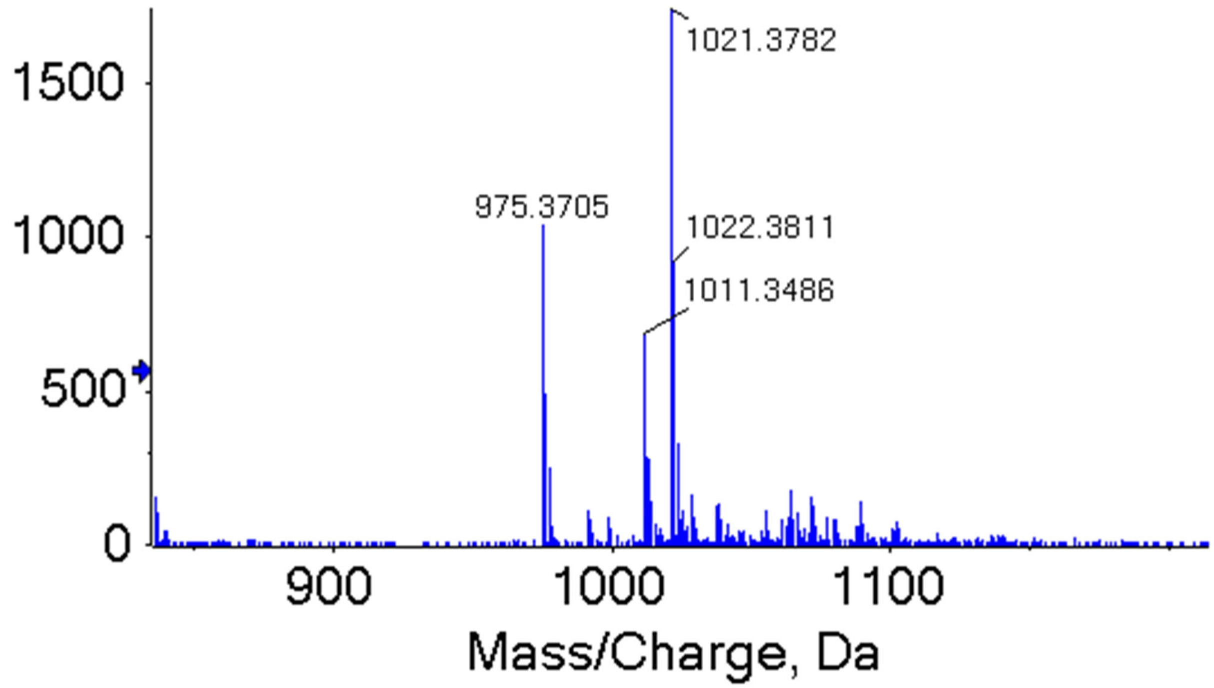

Figure 5. ESI-MS spectrum of compound **1.**

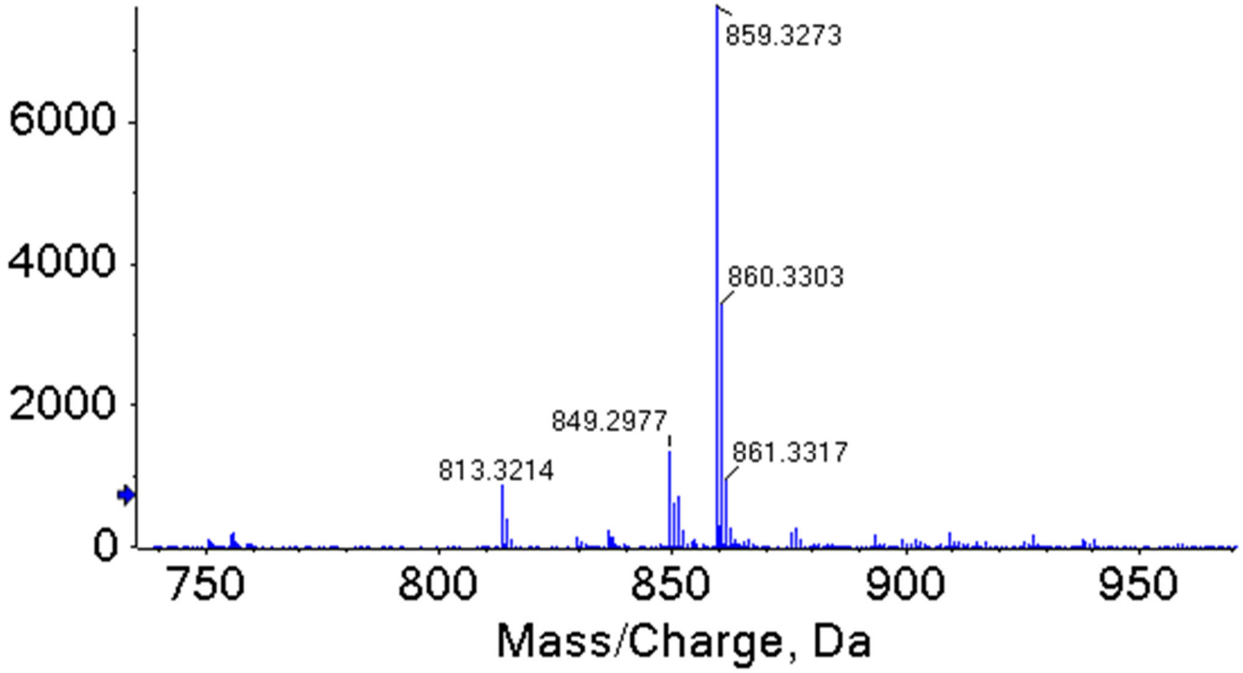

Figure S6. ESI-MS spectrum of compound **2.**
